# Expression of Phospho-ELK1 and Its Prognostic Significance in Urothelial Carcinoma of the Upper Urinary Tract

**DOI:** 10.3390/ijms19030777

**Published:** 2018-03-08

**Authors:** Satoshi Inoue, Hiroki Ide, Kazutoshi Fujita, Taichi Mizushima, Guiyang Jiang, Takashi Kawahara, Seiji Yamaguchi, Hiroaki Fushimi, Norio Nonomura, Hiroshi Miyamoto

**Affiliations:** 1 Department of Pathology & Laboratory Medicine, University of Rochester Medical Center, Rochester, NY 14642, USA; inosts411@gmail.com (S.I.); mizu123shima@gmail.com (T.M.); guiyang_jiang@urmc.rochester.edu (G.J.); takashi_tk2001@yahoo.co.jp (T.K.); 2 James P. Wilmot Cancer Center, University of Rochester Medical Center, Rochester, NY 14642, USA; 3 Department of Pathology and James Buchanan Brady Urological Institute, Johns Hopkins University School of Medicine, Baltimore, MD 21287, USA; h-ide@fc4.so-net.ne.jp; 4 Department of Urology, Osaka University Graduate School of Medicine, Suita 565-0871, Japan; fujita@uro.med.osaka-u.ac.jp (K.F.); nono@uro.med.osaka-u.ac.jp (N.N.); 5 Department of Urology, Osaka General Medical Center, Osaka 558-8558, Japan; yamabu1956@gmail.com; 6 Department of Pathology, Osaka General Medical Center, Osaka 558-8558, Japan; hiroaki-fushimi@gh.opho.jp; 7 Department of Urology, University of Rochester Medical Center, Rochester, NY 14642, USA

**Keywords:** ELK1, immunohistochemistry, upper urinary tract urothelial carcinoma, prognosis, androgen receptor

## Abstract

Using preclinical models, we have recently found that ELK1, a transcriptional factor that activates downstream targets, including *c-fos* proto-oncogene, induces bladder cancer outgrowth. Here, we immunohistochemically determined the expression status of phospho-ELK1, an activated form of ELK1, in upper urinary tract urothelial carcinoma (UUTUC). Overall, phospho-ELK1 was positive in 47 (47.5%; 37 weak (1+) and 10 moderate (2+)) of 99 UUTUCs, which was significantly (*P* = 0.002) higher than in benign urothelium (21 (25.3%) of 83; 17 1+ and 4 2+) and was also associated with androgen receptor expression (*P* = 0.001). Thirteen (35.1%) of 37 non-muscle-invasive versus 34 (54.8%) of 62 muscle-invasive UUTUCs (*P* = 0.065) were immunoreactive for phospho-ELK1. Lymphovascular invasion was significantly (*P* = 0.014) more often seen in phospho-ELK1(2+) tumors (80.0%) than in phospho-ELK1(0/1+) tumors (36.0%). There were no statistically significant associations between phospho-ELK1 expression and tumor grade, presence of concurrent carcinoma in situ or hydronephrosis, or pN status. Kaplan-Meier and log-rank tests revealed that patients with phospho-ELK1(2+) tumor had marginally and significantly higher risks of disease progression (*P* = 0.055) and cancer-specific mortality (*P* = 0.008), respectively, compared to those with phospho-ELK1(0/1+) tumor. The current results thus support our previous observations in bladder cancer and further suggest that phospho-ELK1 overexpression serves as a predictor of poor prognosis in patients with UUTUC.

## 1. Introduction

Upper urinary tract urothelial carcinoma (UUTUC) is a relatively rare disease accounting for only 5–10% of all urothelial carcinomas, whereas urothelial carcinoma of the urinary bladder is a common malignancy, especially in males [1,2]. Due to its preponderance, clinical evidence for bladder cancer has often been applied to decision-making on UUTUC. Indeed, only a few major urological or oncologic associations (e.g., European Association of Urology, Japanese Urological Association) have published a guideline for UUTUC separate from that for bladder cancer [1,2]. More strikingly, there are no prognostic markers for UUTUC available for clinical practice, while alterations in some molecular or genetic factors, which are associated with bladder cancer and serve as its prognosticators, are observed in UUTUC [3,4,5].

ELK1, as a transcription factor, is phosphorylated through activating the mitogen-activated protein kinase (MAPK)/extracellular signal-regulated kinase (ERK) pathways and translocates to the nucleus, leading to the regulation of downstream targets, including a proto-oncogene *c-fos* [6,7], as well as matrix metalloproteinases (MMPs) [8,9] that contribute to tumor cell invasion. Using in vitro and in vivo models for bladder cancer, we have recently found that ELK1 activation correlates with the induction of cell proliferation, migration, and invasion, as well as resistance to cisplatin cytotoxicity [10,11]. Meanwhile, emerging preclinical evidence has indicated a critical role of androgen-mediated androgen receptor (AR) signaling in the development and progression of urothelial cancer [12]. Interestingly, ELK1 appeared to require a functional AR for inducing cell proliferation [10,11]. Indeed, in prostate cancer cells, AR has been shown to function as a co-activator of ELK1 [13]. In surgical specimens, we also demonstrated that ELK1 or phospho-ELK1 (p-ELK1) expression was up-regulated in bladder cancer, compared with non-neoplastic urothelium, and that positivity of p-ELK1, but not ELK1, was associated with the risk of recurrence of non-muscle-invasive tumors (hazard ratio (HR) = 2.829; *P* = 0.056) or cancer-specific mortality in patients with muscle-invasive tumor (HR = 2.693; *P* = 0.021) in a multivariate setting [11].

Thus, ELK1 has been suggested to not only promote urothelial cancer progression, but also function as an important prognosticator for bladder cancer. By contrast, the status of ELK1 expression in UUTUC and its prognostic significance remained uncertain. The aim of this study was to examine the association between p-ELK1 expression and clinicopathological features of UUTUC.

## 2. Results

### 2.1. Immunoreactivity in Benign and Tumor Tissues

**Figure 1:**
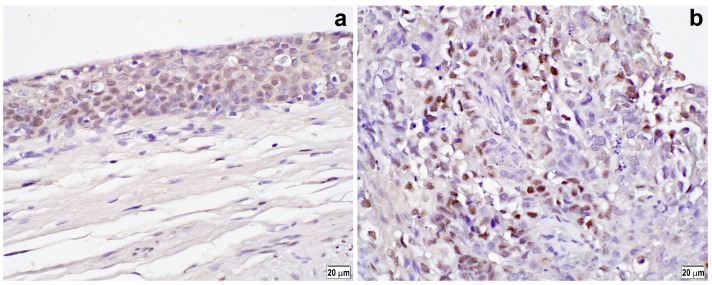
Immunohistochemistry of p-ELK1 in normal urothelial tissue (**a**) and urothelial tumor (**b**). A semi-quantitative analysis of p-ELK1 expression is performed by employing a combination of staining intensity (i.e., weak (**a**), strong (**b**)) and distribution (i.e., percentage of immunoreactive cells). Original magnification: 400×.

Using immunohistochemistry, we investigated the expression of an activated form of ELK1, p-ELK1, in a tissue microarray (TMA) consisting of 99 UUTUC specimens, as well as 83 corresponding normal-appearing urothelial tissue samples. Positive signals for p-ELK1 were detected predominantly in the nuclei of non-neoplastic (Figure 1a) and neoplastic (Figure 1b) epithelial cells. The status of p-ELK1 expression in benign versus tumor tissues is summarized in Table 1. p-ELK1 was positive in 21 (25.3%) of 83 benign urothelial tissues (17 (20.5%) weak (1+) and 4 (4.8%) moderate (2+)) and 47 (47.5%) of 99 urothelial neoplasms (37 (37.4%) 1+ and 10 (10.1%) 2+). Thus, the rate of p-ELK1 positivity was significantly higher in tumors than in benign tissues (*P* = 0.002).

### 2.2. Immunoreactivity and Clinicopathological Features

The status of p-ELK1 expression in UUTUCs according to clinicopathological features is shown in Table 2. The p-ELK1 expression levels tended to be elevated in muscle-invasive tumors, compared with non-muscle-invasive tumors, but they were not statistically different between low-grade and high-grade carcinomas. Lymphovascular invasion was significantly (*P* = 0.014) more often seen in p-ELK1(2+) tumors (8 of 10 (80.0%)) than in p-ELK1(0/1+) tumors (32 of 89 (36.0%)). However, other features, including patient age or gender, tumor laterality, presence of concurrent carcinoma in situ or hydronephrosis, and lymph node involvement, were not significantly associated with p-ELK1 expression. As for tumor site, moderate (2+) p-ELK1 expression was marginally more often (*P* = 0.096) seen in ureteral tumors, compared with renal pelvic tumors. The rates of p-ELK1 positivity in the renal pelvic tumors, ureteral tumors, and bladder tumors were 40.0% (18 of 45), 56.0% (28 of 50), and 65.9% (85 of 129; shown in our previous study [11]), respectively (renal pelvis vs. bladder: *P* = 0.003; ureter vs. bladder: *P* = 0.231).

We then analyzed the relationship between the positivity of p-ELK1 and steroid hormone receptors including AR, estrogen receptor (ER)-α, ERβ, glucocorticoid receptor (GR), and progesterone receptor (PR). Using the same cohort of 99 patients, we reported that AR/ERα/ERβ/GR/PR were positive in 20 (20.2%)/18 (18.2%)/62 (62.6%)/62 (62.6%)/16 (16.2%) UUTUCs, respectively [14]. There was a tendency to show a weak positive correlation (i.e., correlation coefficient (CC) = 0.2–0.4) between p-ELK1 and AR positivity, especially in male tumors (CC = 0.247; *P* = 0.058) (Table 3). Thus, of 52 p-ELK1-negative vs. 47 p-ELK1-positive tumors, 4 (7.7%) vs. 16 (34.0%) were positive for AR (*P* = 0.001). Similarly, of 26 p-ELK1-negative vs. 34 P-ELK1-positive male tumors, 2 (7.7%) vs. 16 (41.2%) were positive for AR (*P* = 0.007). No significant correlations between p-ELK1 and ERα, ERβ, GR, or PR were seen in all 99 tumors, 60 male tumors, and 39 female tumors.

### 2.3. Immunoreactivity and Prognostic Significance

**Figure 2:**
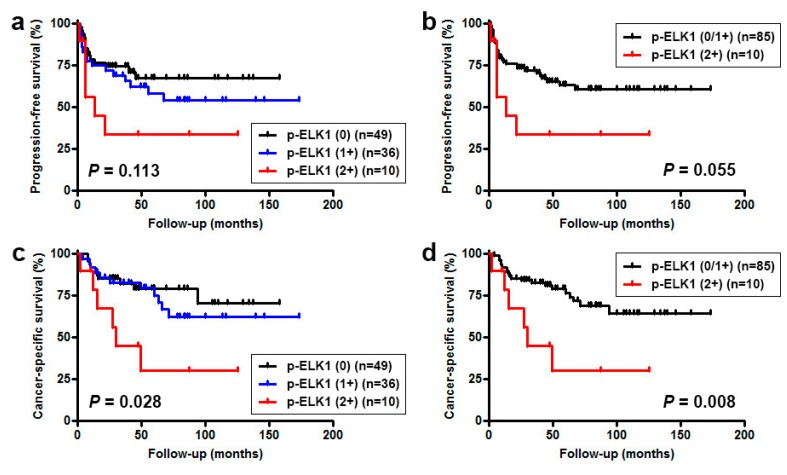
Kaplan-Meier curves for PFS (**a**,**b**) or CSS (**c**,**d**) in 95 patients without metastatic disease, according to the status of p-ELK1 expression.

Next, we investigated possible associations between p-ELK1 expression and patient outcomes. To more accurately assess the role of p-ELK1 expression in disease progression, those with M1 disease (*n* = 4) at the time of nephroureterectomy were excluded from the analyses. Kaplan-Meier and log-rank tests revealed no significant associations between p-ELK1 levels and tumor recurrence in the bladder (0 vs. 1+/2+, *P* = 0.458; 0/1+ vs. 2+, *P* = 0.806). By contrast, moderate p-ELK1 expression was marginally or significantly associated with lower progression-free survival (PFS) (0/1+ vs. 2+, *P* = 0.055; Figure 2a,b), overall survival (OS) (0/1+ vs. 2+, *P* = 0.020; figure not shown), and cancer-specific survival (CSS) (0/1+ vs. 2+, *P* = 0.008; Figure 2c,d) rates. Significant differences in the prognosis were still seen in 81 cases of high-grade tumors (PFS: *P* = 0.042; OS: *P* = 0.022; CSS: *P* = 0.013), but not in 58 cases of muscle-invasive tumors (PFS: *P* = 0.411; OS: *P* = 0.163; CSS: *P* = 0.135).

To determine if p-ELK1 expression status was an independent prognosticator for UUTUC, we then performed multivariate analysis, using the Cox model, for the factors showing *P* < 0.1 in univariate analysis (Table 4). In 95 patients without M1 disease, pT stage and lymphovascular invasion were associated with PFS and/or CSS. However, no significant associations between p-ELK1 expression versus PFS or CSS were found.

## 3. Discussion

The functional role of ELK1, an upstream regulator of the *c-fos* oncogene, in the development and progression of UUTUC remains poorly understood. In the present study, we immunohistochemically determined the expression status of p-ELK1 in UUTUC specimens and its prognostic significance. We first compared the levels of p-ELK1 expression in tumors versus adjacent normal tissues in the upper urinary tract. In accordance with our observations in bladder specimens [11], p-ELK1 expression was significantly up-regulated in tumors, compared with the non-neoplastic urothelium. These results may suggest that ELK1 activation contributes to urothelial tumorigenesis at both the upper and lower urinary tracts. Indeed, we recently found, using an in vitro system, that ELK1 signals were associated with the induction of neoplastic transformation of urothelial cells (Inoue et al., unpublished data).

ELK1 has been implicated in the regulation of cell proliferation, cell cycle control, apoptosis, and cell migration/invasion via, for instance, activation of MAPK/ERK signaling [6,7,15,16]. It also modulates the expression of MMPs [8,9]. Here, we further demonstrated that p-ELK1 overexpression was marginally and significantly associated with muscle invasion and lymphovascular invasion, respectively, in UUTUC. Univariate analysis revealed that p-ELK1 overexpression was also marginally and significantly associated with disease progression and cancer-specific mortality, respectively, in patients with UUTUC. The current findings not only are consistent with those in bladder specimens, indicating the prognostic values of p-ELK1 expression in patients with muscle-invasive tumor [11], but also support our observations in preclinical models suggesting that ELK1 promotes the proliferation, migration, and invasion of bladder cancer cells and activates MMP-2 and MMP-9 [10,11]. Thus, ELK1 activity is suggested to predict the prognosis of UUTUC. However, multivariate analysis did not show statistical significance for p-ELK1 overexpression. In addition, although p-ELK1 positivity in non-muscle-invasive bladder tumors was shown to predict the risk of their recurrence [11], we failed to show an association between p-ELK1 overexpression in UUTUCs and their recurrence in the bladder.

The functional interactions between ELK1 and AR signaling pathways have been documented in prostate cancer cells [13]. We also previously demonstrated activation of ELK1 by androgen-mediated AR signals in bladder cancer cells, as well as a significant association between the expression levels of p-ELK1 and AR in bladder tumor tissue specimens [11]. Moreover, ELK1 inactivation resulted in strong inhibition of the growth of bladder (and prostate) cancer cells only in the presence of an activated AR [10,11,17]. We here showed a marginal association between p-ELK1 and AR expression in UUTUC samples. These findings suggest the involvement of AR signaling in the induction of urothelial cancer progression by ELK1. No significant associations of p-ELK1 expression with that of other steroid hormone receptors, including ERα, ERβ, GR, and PR.

The levels of p-ELK1 expression were higher in ureteral tumors than in renal pelvic tumors, as well as in bladder tumors [11], than in ureteral tumors. Using immunohistochemistry in the same sets of UUTUC and bladder cancer TMAs, we have assessed the expression of various proteins. Interestingly, renal pelvic tumors, compared with ureteral tumors, exhibited lower positive rates of five (out of seven) transcription factors, including AR (11.1% vs. 28.0%, *P* = 0.070) [14], ERβ (51.1% vs. 72.0%, *P* = 0.056) [14], GR (57.8% vs. 68.0%, *P* > 0.1) [14], GATA3 (35.6% vs. 66.0%, *P* = 0.004) [18], and ZKSCAN3 (26.7% vs. 54.0%, *P* = 0.012) [19]. The expression of all of these transcription factors (except ERβ), in addition to p-ELK1, was further up-regulated in bladder tumors, compared with ureteral tumors [20,21,22,23]. The underlying reasons for these findings in the expression of p-ELK1 and other transcription factors in renal pelvic tumors vs. ureteral tumors vs. bladder tumors remain undefined. Of note, the expression patterns of these transcription factors are not well correlated with their functional roles (e.g., tumor suppressive vs. oncogenic) since it has been documented that some promote and others inhibit urothelial cancer outgrowth. However, as we previously suggested [14,24], differences in the anatomic location of renal pelvic/ureteral/bladder tumors and the thickness of the specimens around the tumors might have affected the immunoreactivity for p-ELK1, owing to, for instance, those in the time to complete tissue fixation. Another possibility includes a higher proportion of muscle-invasive disease, where p-ELK1 expression is more likely stronger, in ureteral tumors (33 of 50 (66.0%)) than in renal pelvic tumors (25 of 45 (55.6%)). Meanwhile, the rates of p-ELK1 positivity were similar between benign portions of renal pelvic (11 of 38 (28.9%)) versus ureteral (10 of 41 (24.4%)) urothelium. This may still be because urothelial tissues of both the renal pelvis and ureter are located on the surface of nephroureterectomy specimens when they are opened for gross examination and fixation.

## 4. Materials and Methods

### 4.1. Patients and Tissue Samples

Upon the approval by the institutional review board (IRB #25-2014 at Osaka General Medical Center, Osaka, Japan; Date: 19 June 2013), UUTUC TMA was constructed, as we described previously [25], consisting of dominant tumors and paired normal-appearing urothelial tissues from patients undergoing radical nephroureterectomy. Clinicopathological data of these 99 patients were described previously [14,25] (also see Table 2). There were four cases with metastatic disease where nephroureterectomy was performed mainly for bleeding control. None of the patients had received therapy with anti-cancer drugs or radiation preoperatively.

### 4.2. Immunohistochemistry

Immunohistochemical staining was carried out on the 5 µm sections from the UUTUC TMA, using a primary antibody to p-ELK1 (Ser^383^; sc-8406; Santa Cruz Biotechnology), as we described previously [10,11]. Two pathologists (Guiyang Jiang and Hiroshi Miyamoto), who were blinded to patient identity, independently scored only nuclear staining, using the German Immunoreactive Score (0–12), calculated by multiplying the percentage of immunoreactive cells (0% = 0; 1–10% = 1; 11–50% = 2; 51–80% = 3; 81–100% = 4) by staining intensity (negative = 0; weak = 1; moderate = 2; strong = 3). The scores of 0–1, 2–4, 6–8, and 9–12 were then considered negative (0), weakly positive (1+), moderately positive (2+), and strongly positive (3+), respectively. Cases with discrepancies were re-reviewed simultaneously by the two pathologists until a consensus was reached.

### 4.3. Statistical Analyses

The Fisher’s exact test and Mann–Whitney *U* test were used to assess the statistical significance for categorized variables and those with ordered distribution, respectively. Correlations between variables were determined by the Spearman’s correlation. The rates of recurrence-free survival, PFS, and CSS were calculated by the Kaplan-Meier method, and differences were analyzed by the log-rank test. The Cox proportional hazards model was used for multivariate analysis of prognosticators. *P* values less than 0.05 were considered statistically significant.

## 5. Conclusions

We showed a significant increase in the expression of p-ELK1 in UUTUC, compared with normal-appearing urothelium from each case, implying the involvement of ELK1 signals in the outgrowth of UUTUC. The current results also support our in vitro and in vivo findings in bladder cancer and further suggest that p-ELK1 overexpression serves as a predictor of poor prognosis in patients with UUTUC. Further studies with larger patient cohort are required to validate our observations.

## Figures and Tables

**Table 1:** p-ELK1 expression in non-neoplastic urothelium versus urothelial neoplasm tissue specimens.

Tissue	*n*	p-ELK1 Expression				*P* Value	
		0 (%)	1+ (%)	2+ (%)	3+ (%)	0 vs. 1+/2+/3+	0/1+ vs. 2+/3+
Normal	83	62 (74.7)	17 (20.5)	4 (4.8)	0 (0)	0.002	0.265
Tumor	99	52 (52.5)	37 (37.4)	10 (10.1)	0 (0)		

**Table 2:** Correlations between p-ELK1 expression and clinicopathological profile of the patients.

Parameter	*n*	p-ELK1 Expression			*p* Value	
		0 (%)	1+ (%)	2+ (%)	0 vs. 1+/2+	0/1+ vs. 2+
Age (mean ± SD; years)	99	70.0 ± 9.5	71.9 ± 7.3	68.5 ± 10.9	0.199	0.659
Gender					0.849	0.736
Male	60	28 (46.7)	25 (41.7)	7 (11.7%)		
Female	39	24 (61.5)	12 (30.8)	3 (7.7)		
Laterality					0.548	0.323
Right	43	21 (48.8)	16 (37.2)	6 (14.0)		
Left	56	31 (55.4)	21 (37.5)	4 (7.1)		
Tumor site					0.151 ^a^	0.096 ^a^
Renal pelvis	45	27 (60.0)	16 (35.6)	2 (4.4)		
Ureter	50	22 (44.0)	20 (40.0)	8 (16.0)		
Both	4	3 (75.0)	1 (25.0)	0 (0)		
Tumor grade					0.273	1.000
Low-grade	15	10 (66.7)	4 (26.7)	1 (6.7)		
High-grade	84	42 (50.0)	33 (39.3)	9 (10.7)		
Pathologic stage					0.065 ^b^	0.085 ^b^
pTa	19	13 (68.4)	5 (26.3)	1 (5.3)		
pT1	18	11 (61.1)	7 (38.9)	0 (0)		
NMI (pTa + pT1)	37	24 (64.9)	12 (32.4)	1 (2.7)		
pT2	8	1 (12.5)	6 (75.0)	1 (12.5)		
pT3	48	26 (54.2)	16 (33.3)	6 (12.5)		
pT4	6	1 (16.7)	3 (50.0)	2 (33.3)		
MI (pT2 + pT3 + pT4)	62	28 (45.2)	25 (40.3)	9 (14.5)		
Concurrent CIS					0.768	0.616
No	86	46 (53.5)	32 (37.2)	8 (9.3)		
Yes	13	6 (46.2)	5 (38.5)	2 (15.4)		
Hydronephrosis					0.445 ^c^	1.000 ^c^
No	61	33 (54.1)	25 (41.0)	3 (4.9)		
Yes	20	13 (65.0)	6 (30.0)	1 (5.0)		
Unknown	18	6 (33.3)	6 (33.3)	6 (33.3)		
Lymphovascular invasion					0.227	0.014
No	59	34 (57.6)	23 (39.0)	2 (3.4)		
Yes	40	18 (45.0)	14 (35.0)	8 (20.0)		
Lymph node involvement					0.357 ^d^	0.109 ^d^
pN0	84	41 (48.8)	36 (42.9)	7 (8.3)		
pN1-3	12	8 (66.7)	1 (8.3)	3 (25.0)		
pNx	3	3 (100)	0 (0)	0 (0)		

NMI = non-muscle-invasive; MI = muscle-invasive; CIS = carcinoma in situ. ^a^ Renal pelvis vs. ureter; ^b^ NMI vs. MI; ^c^ No vs. Yes; ^d^ pN0 vs. pN1-3.

**Table 3:** Correlations between p-ELK1 and AR/ERα/ERβ/GR/PR expression.

Patients	*n*	AR		ERα		ERβ		GR		PR	
		CC	*P*	CC	*P*	CC	*P*	CC	*P*	CC	*P*
All cases	99	0.171	0.091	0.076	0.454	0.103	0.312	0.176	0.081	0.054	0.594
Male	60	0.247	0.058	0.175	0.181	0.082	0.535	0.096	0.466	0.055	0.678
Female	39	−0.105	0.525	−0.048	0.770	0.199	0.224	0.262	0.107	0.137	0.407

**Table 4:** Univariate and multivariate analysis of PFS and CSS in 95 patients with UUTUC.

Parameter	Progression-Free Survival						Cancer-Specific Survival					
	Univariate			Multivariate			Univariate			Multivariate		
	HR	95% CI	*P*	HR	95% CI	*P*	HR	95% CI	*P*	HR	95% CI	*P*
Tumor grade	3.858	0.923–16.123	0.064	3.304	0.715–12.877	0.132	6.411	0.868–47.372	0.036	4.953	0.661–37.086	0.119
pT stage ^a^	10.975	3.848–31.306	<0.001	7.750	2.575–23.329	<0.001	17.213	4.055–73.070	<0.001	10.118	2.241–45.680	0.003
LVI	5.701	2.775–11.711	<0.001	2.483	1.125–5.481	0.024	6.712	2.827–15.934	<0.001	2.350	0.888–6.222	0.085
pN stage	4.232	1.738–10.308	0.001	2.494	0.891–6.981	0.082	4.379	1.762–10.884	0.001	1.603	0.605–4.244	0.343
p-ELK1 ^b^	2.291	0.948–5.540	0.066	0.666	0.244–1.820	0.428	3.179	1.279–7.901	0.013	1.131	0.431–2.964	0.802

LVI = lymphovascular invasion; HR = hazard ratio; CI = confidence interval. ^a^ pTa-2 vs. pT3-4; ^b^ 0/1+ vs. 2+.
